# DOAC Score Versus HAS‐BLED and ORBIT for Predicting Bleeding Events in Atrial Fibrillation on Direct Oral Anticoagulants

**DOI:** 10.1002/clc.70315

**Published:** 2026-04-27

**Authors:** Yanfei Guo, Wengen Zhu, Qunfeng Ren

**Affiliations:** ^1^ Department of Cardiovascular Medicine The First People's Hospital of Chenzhou, The First Affiliated Hospital of Xiangnan University Xiangnan University Chenzhou Hunan People's Republic of China; ^2^ Department of Cardiology The First Affiliated Hospital of Sun Yat‐Sen University Guangzhou People's Republic of China; ^3^ Department of Critical Care Medicine The First People's Hospital of Chenzhou, The First Affiliated Hospital of Xiangnan University Xiangnan University Chenzhou Hunan People's Republic of China

**Keywords:** atrial fibrillation, bleeding risk, direct oral anticoagulants, DOAC score

## Abstract

**Background:**

The comparative performance of the DOAC score versus established bleeding risk scores in patients with atrial fibrillation (AF) receiving direct oral anticoagulants (DOACs) remains uncertain. This meta‐analysis evaluated the predictive ability of the DOAC score compared with HAS‐BLED and ORBIT.

**Methods:**

PubMed and Embase were systematically searched to identify studies assessing the predictive performance of the DOAC score in AF patients treated with DOACs. Pooled C‐indices were calculated to compare discrimination. Reclassification metrics (net reclassification improvement [NRI], integrated discrimination improvement [IDI]), calibration analyses, and decision curve analyses (DCA) were synthesized qualitatively.

**Results:**

Nine studies comprising 12 cohorts were included (*n* = 89 688). The DOAC score demonstrated significantly superior discrimination for major bleeding compared with HAS‐BLED (C‐index 0.68 vs. 0.63). No significant differences were observed for intracranial hemorrhage, gastrointestinal bleeding, or clinically relevant non‐major bleeding, nor in comparisons with ORBIT. Reclassification analyses showed heterogeneous findings, with several studies reporting no incremental benefit of the DOAC score, although one large cohort demonstrated improved NRI and IDI over HAS‐BLED. Calibration analyses revealed good performance across scores, though both HAS‐BLED and DOAC tended to overestimate bleeding risk in high‐risk groups. DCA suggested variable but occasionally greater net benefit of the DOAC score at clinically relevant risk thresholds.

**Conclusions:**

The DOAC score provides modest but statistically significant improvement in predicting major bleeding compared with HAS‐BLED, with comparable performance to ORBIT. However, reclassification, calibration, and clinical utility vary across settings, underscoring the need for further prospective validation.

## 1 Introduction

Atrial fibrillation (AF) is the most prevalent sustained cardiac arrhythmia and is strongly associated with an elevated risk of stroke and systemic embolism, rendering long‐term anticoagulation essential for a substantial proportion of patients [1, 2]. The advent of direct oral anticoagulants (DOACs) has improved the safety, convenience, and overall clinical applicability of anticoagulation compared with vitamin K antagonists (VKAs); however, bleeding complications remain a major determinant of clinical outcomes and continue to influence treatment selection and monitoring strategies. Consequently, clinicians frequently rely on validated bleeding risk stratification tools to support individualized decision‐making, refine the balance between thromboembolic protection and hemorrhagic risk, and guide follow‐up intensity. Among these tools, the HAS‐BLED score, originally developed and validated in VKA‐treated cohorts [3, 4], remains widely used despite its limited calibration and only modest discriminatory performance in contemporary DOAC‐treated populations, as consistently demonstrated in several meta‐analyses [5, 6, 7].

Recognizing these limitations, increasing attention has been directed toward the development of bleeding risk models specifically tailored to DOAC‐treated cohorts [7, 8]. The DOAC score was derived from the RE‐LY trial and subsequently validated using the GARFIELD‐AF registry, followed by external validation in COMBINE‐AF and multiple real‐world populations across diverse healthcare settings [9, 10, 11, 12, 13, 14, 15, 16, 17]. In its derivation and early validation studies, the DOAC score exhibited superior discriminative performance compared with HAS‐BLED and demonstrated improved risk stratification in routine clinical practice [9]. However, the reproducibility of its predictive advantage has been inconsistent, with several contemporary cohorts reporting only modest discrimination for both the DOAC score and HAS‐BLED, and in some cases showing no statistically significant difference between the two [13, 16]. Furthermore, findings from a large multicenter Asian cohort showed that, although the overall predictive accuracy of both scores remained moderate, the DOAC score achieved a slightly but statistically significant improvement in C‐index, along with better net reclassification metrics, suggesting potential utility in populations with distinct demographic or clinical risk profiles [17]. These observations highlight the possibility that bleeding risk prediction may be influenced by ethnic, genetic, or practice‐pattern differences, underscoring the need for broader evidence synthesis.

The ORBIT bleeding score [18, 19], which incorporates clinically relevant variables such as age, anemia, impaired renal function, prior bleeding events, and concomitant antiplatelet use, has also gained traction as an alternative risk stratification tool in anticoagulated AF populations. Prior meta‐analyses including both DOAC‐ and VKA‐treated patients have generally demonstrated that ORBIT and HAS‐BLED yield comparable predictive performance for major bleeding, with no consistent or clinically meaningful superiority observed for either score [5, 20]. Given the heterogeneity and inconsistency across published studies, a comprehensive meta‐analytic synthesis is warranted to more clearly define the comparative predictive accuracy of the DOAC score relative to HAS‐BLED and ORBIT in AF patients treated with DOACs.

## 2 Methods

### 2.1 Literature Search

We comprehensively searched the PubMed and Embase electronic databases from inception to November 2025 to identify relevant studies reporting the performance of the DOAC score in predicting bleeding events among patients with AF receiving DOACs. The following keywords were used in the search strategies: (1) *atrial fibrillation* AND (2) *direct oral anticoagulants* OR *DOACs* OR *non–vitamin K antagonist oral anticoagulants* OR *NOACs* OR *dabigatran* OR *rivaroxaban* OR *apixaban* OR *edoxaban* AND (3) *DOAC score* OR *bleeding score* OR *risk score*. We did not include other risk‐prediction tools in the search to maintain focus on the DOAC score. In addition, reference lists of previous reviews and eligible articles were screened to identify additional studies. Only studies published in English were included. Supporting Information S1: Table 1 shows the search strategies of this meta‐analysis.

### 2.2 Eligibility Criteria

We included studies that met the following criteria: (a) adult patients with nonvalvular AF receiving DOACs; (b) studies reporting the diagnostic or predictive performance of the DOAC score in comparison with the HAS‐BLED and/or ORBIT scores; (c) studies evaluating major bleeding or other bleeding outcomes, including major bleeding or clinically relevant nonmajor bleeding (CRNMB), intracranial hemorrhage, or gastrointestinal bleeding; and (d) studies providing at least one of the following metrics: C‐index, calibration data, reclassification measures such as the net reclassification improvement (NRI) or integrated discrimination improvement (IDI), or decision curve analysis.

Studies conducted exclusively in patients with venous thromboembolism or other non‐AF populations were excluded, even if they evaluated the DOAC score. We also excluded studies with insufficient or non‐original data, including reviews, case reports, comments, editorials, letters, conference abstracts, and other non‐peer‐reviewed publications.

### 2.3 Variables in HAS‐BLED, ORBIT, and DOAC Scores

The three bleeding risk scores incorporate distinct yet overlapping sets of clinical indicators. The HAS‐BLED score [3] assesses hypertension, abnormal renal or liver function, stroke history, prior bleeding, labile international normalized ratio (INR), age > 65, and concomitant drug/alcohol use. The ORBIT score [18] focuses on a narrower set of factors: age ≥ 75, anemia, bleeding history, renal insufficiency, and antiplatelet use. In contrast, the DOAC score [9] includes a more comprehensive profile, featuring stratified age groups, detailed renal function categories, low body weight, diabetes, hypertension, specific types of antiplatelet and non‐steroidal anti‐inflammatory drug (NSAID) use, as well as bleeding history and liver disease, resulting in a broader assessment of a patient's bleeding risk profile.

### 2.4 Study Selection and Data Extraction

Two reviewers independently screened all identified studies according to the predefined eligibility criteria. Studies were selected through an initial title and abstract review followed by full‐text assessment. Any discrepancies were resolved through discussion or consultation with a third reviewer.

Data were extracted from all eligible studies using a standardized form. The following information was collected: first author, year of publication, study design, data source, baseline characteristics of the study population (age, sex distribution, sample size, type of DOAC used), bleeding outcomes, bleeding definitions, comparator scores (HAS‐BLED and/or ORBIT), and duration of follow‐up.

### 2.5 Quality Assessment

The methodological quality and risk of bias of the included studies were assessed using the Prediction Model Risk of Bias Assessment Tool (PROBAST; www.probast.org) [21]. Specifically, the PROBAST + AI tool was applied, which is structured around four key domains (participants, predictors, outcome, and analysis) and is designed for the critical appraisal of prediction model studies. Each domain was evaluated for risk of bias as well as for concerns regarding applicability.

### 2.6 Statistical Analysis

Statistical heterogeneity among the included studies was evaluated using the Cochrane *Q* test and the *I*² statistic. Significant heterogeneity was defined as a *p* < 0.1 in the *Q* test or an *I*² > 50%. For model discrimination, C‐indexes and their corresponding 95% confidence intervals (CIs) were extracted and pooled using a random‐effects model with the inverse variance method. *Z*‐statistics were calculated to compare the pooled C‐indexes of the DOAC score with those of the HAS‐BLED and ORBIT scores. For the primary outcome of major bleeding, subgroup analyses and sensitivity analyses were performed based on available study characteristics when applicable. Publication bias was assessed visually by funnel plots, with notable asymmetry indicating potential bias.

In addition to discrimination analysis, narrative synthesis was conducted for reclassification metrics, including NRI and IDI, to evaluate incremental predictive value. Calibration data were reviewed to determine the agreement between predicted risks and observed bleeding events. Decision curve analysis evaluated the clinical net benefit of using a risk score across various decision thresholds.

All statistical analyses were performed using Review Manager version 5.4 (The Cochrane Collaboration, Nordic Cochrane Centre, Copenhagen, Denmark). A two‐sided *p* < 0.05 was considered statistically significant.

## 3 Results

### 3.1 Study Selection

**Figure 1:**
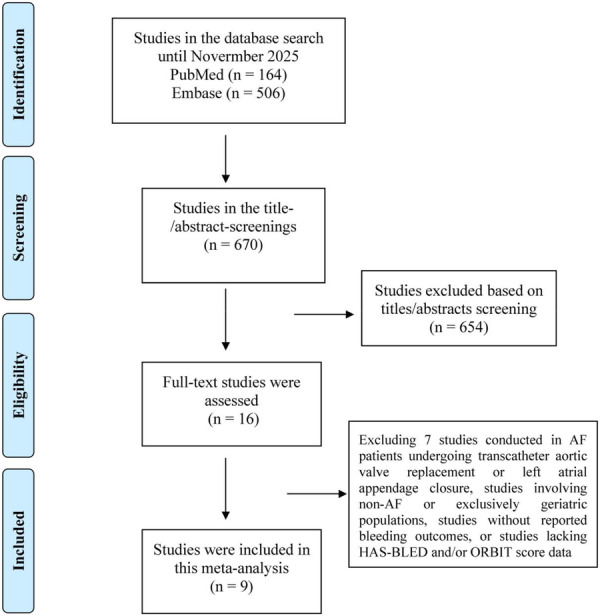
Flowchart of the study retrieval and screening process of this meta‐analysis.

The flow diagram outlining the study identification and screening process is presented in Figure 1. A total of 670 records were retrieved through comprehensive searches of the PubMed and Embase databases. After title and abstract screening, 16 articles were selected for full‐text evaluation. Seven studies [22, 23, 24, 25, 26, 27, 28] were subsequently excluded based on predefined eligibility criteria, including studies enrolling patients with AF undergoing transcatheter aortic valve replacement or left atrial appendage closure, studies limited to non‐AF or exclusively geriatric populations, those lacking bleeding outcome data, or those not reporting HAS‐BLED and/or ORBIT scores. Ultimately, nine studies comprising 12 cohorts [9, 10, 11, 12, 13, 14, 15, 16, 17] met all inclusion criteria and were incorporated into the final meta‐analysis. The DOAC score was originally derived from the RE‐LY trial and subsequently validated across COMBINE‐AF and several large real‐world data sets [9, 10, 11, 12, 13, 14, 15, 16, 17].

### 3.2 Baseline Characteristics

Baseline characteristics of the included cohorts are summarized in Table 1. The study populations consisted of adult patients with AF treated with dabigatran, rivaroxaban, apixaban, or edoxaban. Sample sizes ranged from 313 to 25 586 participants (total *n* = 89 688). Across studies, mean or median ages generally ranged from 70 to 76 years, with women comprising approximately 28%−48% of participants. Major bleeding, most often defined according to International Society on Thrombosis and Hemostasis criteria, was the primary outcome, although several studies additionally assessed CRNMB, intracranial hemorrhage, or gastrointestinal bleeding. Follow‐up durations varied between 1 and 5 years. All included studies compared the discriminative performance of the DOAC score with the HAS‐BLED score, and several also evaluated the ORBIT score. The cohorts originated from multiple international registries and cohorts across Asia, Europe, and North America.

**Table 1:** Summary of baseline characteristics from the included studies in this meta‐analysis.

Study (author‐year)	Data sources	Age (mean or median; years)	Females (%)	Sample size (*n*)	DOAC types	Bleeding events	Bleeding definitions	Bleeding scores for analysis	Follow‐up (years)
Aggarwal‐2023	The RE‐LY development cohort; global; 2005–2007	MB: 70.1 No MB: 75.8	36.6	5684	Dabigatran	MB	ISTH	DOAC versus HAS‐BLED	1.74
	GARFIELD‐AF registry; global; 2013–2016	MB: 78.0 No MB: 720	43.9	12 296	Dabigatran, rivaroxaban, apixaban, edoxaban	MB	ISTH	DOAC versus HAS‐BLED	1.0
	COMBINE‐AF cohort; global	NA	NA	25 586	Rivaroxaban, apixaban, edoxaban	MB	ISTH	DOAC versus HAS‐BLED	1.0
	RAMQ cohort; Canada; 2011−2017	NA	NA	11 945	Rivaroxaban, apixaban	MB	ICD codes	DOAC versus HAS‐BLED	1.0
Abu‐Assi‐2024^a^	CardioCHUVI‐AF registry; Spain; 2014–2018	NA	NA	1484	Dabigatran, rivaroxaban, apixaban, edoxaban	MB	ISTH	DOAC versus HAS‐BLED/ORBIT	3.5
Akao‐2024^a^	J‐RISK AF study; Japan; 2016−2018	70.0	28.1	1562	Dabigatran, rivaroxaban, apixaban, edoxaban	MB	ISTH	DOAC versus HAS‐BLED/ORBIT	2.0
Fan‐2024	Ningbo university; China; 2017−2022	71.7	41.2	2532	Dabigatran, rivaroxaban	MB; ICH; GIB	ISTH	DOAC versus HAS‐BLED/ORBIT	1.0
Mei‐2024	Prospective, observational registry held in Europe; 2013−2016	69.0	39.0	2834	DOACs, Not specified	MB	ISTH	DOAC versus HAS‐BLED	2.0
Soler‐Espejo‐2025^a^	Prospective observational cohort (MAFP‐III); Spain; 2016–2021	NA	NA	2209	Dabigatran, rivaroxaban, apixaban, edoxaban	MB; Major bleeding/CRNMB	ISTH	DOAC versus HAS‐BLED/ORBIT	2.0
Nishiyama‐2025	KiCS‐AF; Japan; 2012−2018	70.0	32.1	2101	Dabigatran, rivaroxaban, apixaban, edoxaban	MB	ISTH	DOAC versus HAS‐BLED/ORBIT	5.0
Almalbis‐2025	Hospital Canselor Tuanku Muhriz; Malaysia; 2015−2020	70.9	48.2	313	Rivaroxaban, apixaban	Major bleeding/CRNMB	ISTH	DOAC versus HAS‐BLED/ORBIT	1.0
Chan‐2025	Chang Gung Research Database; Taiwan; 2012−2021	75.9	41.0	21 142	Dabigatran, rivaroxaban, apixaban, edoxaban	MB; ICH; GIB	ISTH	DOAC versus HAS‐BLED	1.0

Abbreviations: COMBINE‐AF, A Collaboration Between Multiple Institutions to Better Investigate Non‐Vitamin K Antagonist Oral Anticoagulant Use in Atrial Fibrillation; CRNMB, clinically relevant nonmajor bleeding; DOACs, direct oral anticoagulants; GARFIELD‐AF, Global Anticoagulant Registry in the Field‐Atrial Fibrillation; GIB, gastrointestinal bleeding; HAS‐BLED, Hypertension, Abnormal liver/renal function, Stroke, Bleeding history or predisposition, Labile international normalized ratio, Elderly, Drugs/alcohol concomitantly; ICD, International Classification of Diseases; ICH, intracranial hemorrhage; ISTH, International Society of Thrombosis and Hemostasis; MB, major bleeding; NA, not available; ORBIT, outcomes Registry for Better Informed Treatment of Atrial Fibrillation; RAMQ, Quebec Régie de l'Assurance Maladie du Québec and Med‐Echo Administrative Databases; RE‐LY, Randomized Evaluation of Long‐Term Anticoagulation Therapy.

a Subgroup patients with DOACs were included in this meta‐analysis.

Risk of bias and applicability were evaluated using the PROBAST + AI tool (Supporting Information S1: Table 2). Most studies were rated as having a low risk of bias and low applicability concerns. The study by Mei et al. showed a high risk of bias but low applicability concerns, whereas the study by Almalbis et al. was judged to have both a high risk of bias and high concerns regarding applicability.

### 3.3 Discrimination Between DOAC Score Versus HAS‐BLED and ORBIT

**Figure 2:**
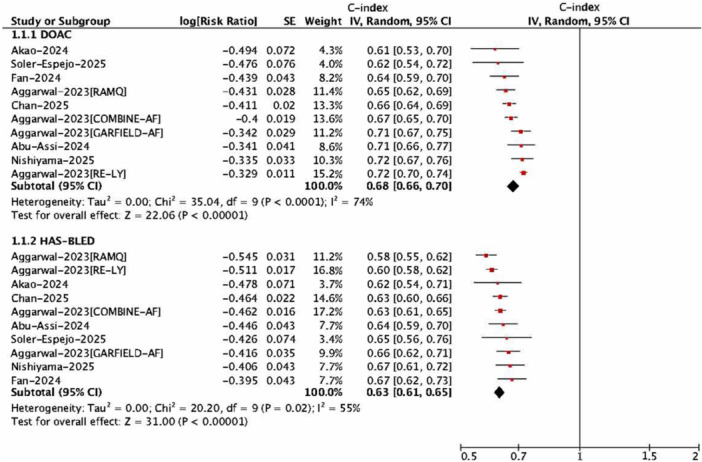
Discrimination between DOAC versus HAS‐BLED scores. DOAC, direct oral anticoagulant; HAS‐BLED, Hypertension, Abnormal liver/renal function, Stroke, Bleeding history or predisposition, Labile international normalized ratio, Elderly, Drugs/alcohol concomitantly.

As shown in Table 2, pooled analyses demonstrated that the DOAC score exhibited significantly better discrimination for major bleeding compared with the HAS‐BLED score (*p* = 0.00053), yielding a pooled C‐index of 0.68 (95% CI, 0.66–0.70) versus 0.63 (95% CI, 0.61–0.65) (Figure 2). For other bleeding outcomes, however, no significant differences were observed between the two scores: major bleeding or CRNMB (0.62 [0.56–0.68] vs. 0.60 [0.51–0.71]), intracranial hemorrhage (0.61 [0.52–0.71] vs. 0.63 [0.52–0.76]), and gastrointestinal bleeding (0.67 [0.61–0.73] vs. 0.65 [0.62–0.68]).

**Table 2:** Comparison of C‐indexes and 95% CIs between the DOAC score and the HAS‐BLED and ORBIT scores.

	Major bleeding	Major bleeding or CRNMB	Intracranial hemorrhage	Gastrointestinal bleeding
DOAC versus HAS‐BLED				
No. of studies	6	2	2	2
Range in C‐index: DOAC	0.61−0.72	0.50−0.66	0.56−0.66	0.63−0.69
Pooled C‐index: DOAC	0.68 (0.66−0.70)	0.62 (0.56−0.68)	0.61 (0.52−0.71)	0.67 (0.61−0.73)
Range in C‐index: HAS‐BLED	0.58−0.67	0.42−0.64	0.58−0.70	0.64−0.65
Pooled C‐index: HAS‐BLED	0.63 (0.61−0.65)	0.60 (0.51−0.71)	0.63 (0.52−0.76)	0.65 (0.62−0.68)
Z‐statistic	3.466	0.336	−0.256	0.584
*p* value	0.00053	0.736	0.798	0.558
DOAC versus ORBIT				
No. of studies	5	2	—	—
Range in C‐index: DOAC	0.61−0.72	0.50−0.66		
Pooled C‐index: DOAC	0.67 (0.63−0.71)	0.62 (0.56−0.68)	—	—
Range in C‐index: ORBIT	0.61−0.71	0.58−0.68		
Pooled C‐index: ORBIT	0.66 (0.63−0.70)	0.66 (0.62−0.71)	—	—
Z‐statistic	0.369	−1.045		
*p* value	0.712	0.296		

Abbreviations: CI, confidence interval; CRNMB, clinically relevant nonmajor bleeding; HAS‐BLED, Hypertension, Abnormal liver/renal function, Stroke, Bleeding history or predisposition, Labile international normalized ratio, Elderly, Drugs/alcohol concomitantly; ORBIT, Outcomes Registry for Better Informed Treatment of Atrial Fibrillation.

**Figure 3:**
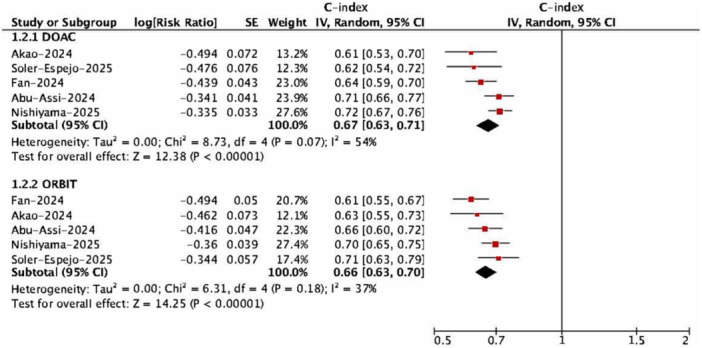
Discrimination between DOAC versus ORBIT scores. DOAC, direct oral anticoagulant; ORBIT, Outcomes Registry for Better Informed Treatment of Atrial Fibrillation.

In comparison with the ORBIT score, the discriminative performance of the DOAC score was similar. For major bleeding, pooled C‐indices were 0.67 (95% CI, 0.63–0.71) for the DOAC score and 0.66 (95% CI, 0.63–0.70) for ORBIT (Figure 3). For the composite outcome of major bleeding or CRNMB, the corresponding values were 0.62 (95% CI, 0.56–0.68) versus 0.66 (95% CI, 0.62–0.71), with no statistically significant differences.

### 3.4 Reclassification Between DOAC Score Versus HAS‐BLED and ORBIT

Reclassification analyses yielded heterogeneous findings across studies. In the cohort analyzed by Fan et al., neither the DOAC score nor the HAS‐BLED or ORBIT scores showed significant improvement in reclassification, as indicated by nonsignificant NRI and IDI metrics, suggesting no incremental value of the DOAC score over established tools in this Chinese AF population. Mei et al. similarly reported nonsignificant NRI and IDI values at both 1‐ and 2‐year follow‐up intervals, indicating no advantage of the DOAC score over HAS‐BLED.

In contrast, Soler‐Espejo et al. observed a borderline significant decrement in reclassification with the DOAC score relative to the ORBIT score for major bleeding, with an NRI of –11.2% (95% CI, –26.2% to 0.8%; *p* = 0.093) and an IDI of –1.2% (95% CI, –2.5% to 0%; *p* = 0.004), while no improvement was noted compared with HAS‐BLED. Conversely, Chan et al. reported a significant reclassification benefit of the DOAC score relative to HAS‐BLED in a large Taiwanese cohort, with an NRI of 25.7% (95% CI, 18.1%–33.2%; *p* < 0.001) and an IDI of 0.26% (95% CI, 0.17%–0.35%; *p* < 0.001).

### 3.5 Calibration Between DOAC Score Versus HAS‐BLED and ORBIT

Calibration assessments also varied across studies. Fan et al. reported that HAS‐BLED demonstrated superior calibration compared with the DOAC and ORBIT scores, reflecting closer agreement between predicted and observed bleeding risks. Mei et al. found good calibration for both the DOAC and HAS‐BLED scores in lower‐risk strata, although the DOAC score overestimated bleeding risk among high‐risk patients. Abu‐Assi et al. reported acceptable overall calibration for all three risk models, though HAS‐BLED performed marginally worse than the DOAC and ORBIT scores. Chan et al. observed that both HAS‐BLED and DOAC scores were well‐calibrated in low‐ to intermediate‐risk groups but overestimated major bleeding risk in high and very‐high‐risk categories.

### 3.6 Clinical Utility Between DOAC Score Versus HAS‐BLED and ORBIT

Decision curve analysis demonstrated notable variability among studies. Fan et al. reported that HAS‐BLED yielded the greatest net clinical benefit for predicting major bleeding and intracranial hemorrhage. Mei et al. found no discernible difference in net benefit between HAS‐BLED and DOAC scores. In contrast, Abu‐Assi et al. demonstrated that the DOAC score provided superior net benefit across a wide spectrum of clinically relevant decision thresholds compared with both HAS‐BLED and ORBIT. Similarly, Chan et al. showed that the DOAC score outperformed HAS‐BLED across bleeding risk thresholds of approximately 2%−5%.

### 3.7 Subgroup and Sensitivity Analyses

Sensitivity analyses performed by sequentially excluding each included study showed stable results, confirming the robustness of the primary findings. Additional analyses excluding the RE‐LY derivation cohort and restricting the data set to validation cohorts yielded results consistent with the overall analysis. Subgroup analyses stratified by region (Asia, Europe, and North America) and follow‐up duration (≥ 2 vs. < 2 years) demonstrated no significant interactions, as all interaction *p*‐values exceeded 0.05.

### 3.8 Publication Bias

Visual inspection of funnel plots demonstrated symmetrical distribution for comparisons of DOAC versus HAS‐BLED (Supporting Information S1: Figure 1) and DOAC versus ORBIT (Supporting Information S1: Figure 2), suggesting minimal evidence of publication bias.

## 4 Discussion

In this comprehensive meta‐analysis including 9 studies and 12 cohorts totaling 89 688 patients, the DOAC score demonstrated a statistically significant but modest improvement in discriminative performance for predicting major bleeding compared with the HAS‐BLED score, while showing overall comparable performance with the ORBIT score. These findings reinforce the emerging concept that bleeding risk in DOAC‐treated AF patients may be more accurately captured by models specifically developed from and validated in DOAC‐era cohorts [9, 11, 23, 28, 29, 30]. Despite this incremental improvement, the absolute degree of discrimination achieved by the DOAC score remained moderate, and reclassification, calibration, and decision curve analyses revealed substantial heterogeneity across clinical settings. This underscores the complexity of bleeding prediction in contemporary AF populations, where improvements in anticoagulant safety, variation in patient characteristics, and differences in prescribing practices introduce considerable variability that may limit the generalizability of any single risk model.

Our findings align with prior evidence demonstrating the limited performance of HAS‐BLED in DOAC‐treated cohorts [5, 6], despite its longstanding clinical utility and endorsement in major international guidelines [1, 2]. As HAS‐BLED was derived in VKA‐treated patients [3], several of its predictors, particularly labile INR, have reduced relevance in the DOAC era. Although the ORBIT score was developed in mixed cohorts and incorporates fewer variables, its discriminative performance remained comparable to HAS‐BLED [6, 20]. The DOAC score [9] was designed to integrate DOAC‐specific bleeding determinants, such as stratified renal function, low body weight, and detailed antiplatelet/NSAID exposure, potentially explaining its observed advantage over HAS‐BLED in predicting major bleeding. However, this advantage was not consistently replicated across all bleeding endpoints (e.g., intracranial hemorrhage, gastrointestinal bleeding), suggesting that even DOAC‐tailored predictors may inadequately capture the multifactorial nature of bleeding risk.

Reclassification analyses demonstrated marked heterogeneity across the included studies, highlighting potential differences in risk distributions and baseline hazards across populations. Notably, the large Taiwanese cohort reported substantial NRI and IDI improvements with the DOAC score, suggesting potential benefits in Asian populations where body size, genetic determinants of drug sensitivity, and prescribing patterns differ from Western cohorts [17]. In contrast, multiple European cohorts [13, 14] demonstrated no incremental value of the DOAC score over HAS‐BLED or ORBIT, reinforcing concerns regarding inconsistent external validity. Calibration analyses similarly showed variability, with the DOAC score tending to overestimate bleeding risk in high‐risk strata in several cohorts [13, 17]. These calibration limitations may undermine clinical applicability, particularly given the low absolute bleeding risk observed in large contemporary DOAC registries.

Decision curve analyses revealed that the clinical utility of the DOAC score varied across cohorts and decision thresholds. In some cohorts, notably Abu‐Assi et al. [10] and Chan et al. [17], the DOAC score conferred enhanced net benefit, suggesting potential value in settings where clinical thresholds for intensifying monitoring or modifying therapy are tightly linked to individualized bleeding risk estimation. However, Mei et al. [13] demonstrated no meaningful difference between scores, raising the possibility that contextual factors, such as underlying comorbidity burden, regional DOAC prescribing preferences, or differential availability of follow‐up, may influence the realized clinical utility of risk stratification tools.

Taken together, these findings highlight that although the DOAC score modestly improves bleeding risk prediction compared with HAS‐BLED and performs similarly to ORBIT, its clinical advantage is neither universal nor robust across diverse populations and bleeding outcomes. Importantly, none of the evaluated scores achieved high discriminative performance, consistent with long‐standing challenges in bleeding risk modeling where the stochastic nature of bleeding events, competing risks, and time‐varying exposures complicate prediction. Current international guidelines emphasize that bleeding risk scores should inform, rather than dictate, anticoagulation decisions, and should primarily identify modifiable risk factors rather than serve as a basis for withholding anticoagulation therapy [1, 2]. Our findings support this principle and underscore the need for next‐generation bleeding risk models incorporating dynamic, longitudinal, and biomarker‐informed predictors, potentially using machine‐learning approaches under rigorous methodological frameworks [8, 31, 32, 33, 34, 35].

## 5 Limitations

This meta‐analysis has several limitations that merit consideration. First, although the included cohorts were derived from diverse geographic regions, inherent heterogeneity in patient demographics, comorbidity burden, DOAC selection, and healthcare systems may have influenced bleeding risks and the performance of prediction models. Second, despite rigorous methodology, the number of eligible studies evaluating the DOAC score remains limited, particularly for outcomes other than major bleeding, reducing statistical power for these secondary endpoints. Third, reclassification, calibration, and decision curve metrics could not be meta‐analyzed due to inconsistent reporting and methodological variability, necessitating qualitative synthesis that may introduce interpretive subjectivity. Fourth, most included studies were observational, and although the risk of bias was generally low, residual confounding cannot be excluded. Finally, because risk scores were applied based on baseline characteristics only, dynamic changes in renal function, hemoglobin, concomitant medications, and frailty were not incorporated. These time‐varying variables are clinically relevant predictors of bleeding and may influence the performance of all three scores. As DOAC‐treated patients become older and more multimorbid, reliance on static baseline scores may increasingly limit predictive accuracy.

## 6 Conclusion

The DOAC score provided modest but statistically significant improvement in the prediction of major bleeding compared with HAS‐BLED in patients with AF receiving DOACs, while demonstrating performance comparable to the ORBIT score. However, reclassification, calibration, and clinical utility varied considerably across populations, and no model achieved high discrimination. These findings support the use of bleeding scores as supportive tools for clinical risk assessment rather than as determinants of anticoagulation decisions. Further large‐scale, prospective, externally validated studies are needed to refine bleeding risk assessment in the DOAC era and improve individualized care.

## Author Contributions

Yanfei Guo, Wengen Zhu, and Qunfeng Ren finished all the work.

## Ethics Statement

The authors have nothing to report.

## Consent

The authors have nothing to report.

## Conflicts of Interest

The authors declare no conflicts of interest.

## Supporting information


**Supporting file :** clc70315‐sup‐0001‐Supplemental_files.docx.

## Data Availability

The data supporting the findings of this study are available upon reasonable request from the corresponding author.
