# Supplementary material for: DOAC Score Versus HAS‐BLED and ORBIT for Predicting Bleeding Events in Atrial Fibrillation on Direct Oral Anticoagulants
Source: Clin Cardiol. 2026 Apr 27;49(5):e70315. doi: 10.1002/clc.70315 (PMC13112412; doi:10.1002/clc.70315)
Supplement: Supplementary file 1 — Supporting file : clc70315‐sup‐0001‐Supplemental_files.docx. [file CLC-49-e70315-s001.docx]

**Supplemental Table 1. Literature search of this meta-analysis**

|  | **Pubmed** | **Embase** |
| --- | --- | --- |
| **Items** | (  "Anticoagulants"[Mesh] OR anticoagulant therapy[tiab] OR oral anticoagulants[tiab]  OR direct oral anticoagulants[tiab] OR DOAC[tiab] OR DOACs[tiab]  OR NOAC[tiab] OR NOACs[tiab] OR non-vitamin K antagonist oral anticoagulants[tiab]  OR novel oral anticoagulants[tiab]  OR factor Xa inhibitors[tiab] OR thrombin inhibitors[tiab]  OR dabigatran[tiab] OR rivaroxaban[tiab] OR apixaban[tiab]  OR edoxaban[tiab]  )  AND  (atrial fibrillation[tiab] OR AF[tiab])  AND  (bleeding[tiab] OR hemorrhage[tiab] OR major bleeding[tiab]  OR bleeding events[tiab] OR bleeding risk[tiab])  AND  ("DOAC score"[tiab] OR DOAC-score[tiab] OR bleeding score[tiab]  OR risk score[tiab] OR predictive score[tiab]) | (  'anticoagulant agent'/exp OR 'anticoagulant agent':ti,ab  OR 'anticoagulant'/exp OR 'anticoagulant':ti,ab  OR 'oral anticoagulant':ti,ab OR 'oral anticoagulants':ti,ab  OR 'direct oral anticoagulant':ti,ab OR 'direct oral anticoagulants':ti,ab  OR 'doac':ti,ab OR 'doacs':ti,ab  OR 'noac':ti,ab OR 'noacs':ti,ab  OR 'non-vitamin k antagonist oral anticoagulant':ti,ab  OR 'non-vitamin k antagonist oral anticoagulants':ti,ab  OR 'dabigatran'/exp OR 'dabigatran':ti,ab  OR 'dabigatran etexilate':ti,ab OR 'pradaxa':ti,ab  OR 'rivaroxaban'/exp OR 'rivaroxaban':ti,ab OR 'xarelto':ti,ab  OR 'apixaban'/exp OR 'apixaban':ti,ab OR 'eliquis':ti,ab  OR 'edoxaban'/exp OR 'edoxaban':ti,ab OR 'lixiana':ti,ab OR 'savaysa':ti,ab  OR 'betrixaban'/exp OR 'betrixaban':ti,ab OR 'bevyxxa':ti,ab  OR 'direct thrombin inhibitor':ti,ab  OR 'direct factor xa inhibitor':ti,ab  OR 'factor xa inhibitor':ti,ab  )  AND  ('atrial fibrillation'/exp OR 'atrial fibrillation':ti,ab OR 'af':ti,ab)  AND  ('bleeding'/exp OR bleeding:ti,ab  OR 'hemorrhage'/exp OR hemorrhage:ti,ab  OR 'major bleeding':ti,ab  OR 'bleeding event':ti,ab OR 'bleeding events':ti,ab  OR 'bleeding risk':ti,ab)  AND  ('doac score':ti,ab OR doac-score:ti,ab  OR 'bleeding score':ti,ab  OR 'risk score':ti,ab  OR 'predictive score':ti,ab) |
| **No.** | 164 | 506 |

**Supplemental Table 2. PROBAST+AI: risk of bias and applicability assessment**

| **Study(author-year)** | **Risk of bias** | **Applicability** |
| --- | --- | --- |
| Aggarwal-2023 | Low | Low concern |
| Abu-Assi-2024 | Low | Low concern |
| Akao-2024 | Low | Low concern |
| Fan-2024 | Low | Low concern |
| Mei-2024 | High | Low concern |
| Soler-Espejo-2025 | Low | Low concern |
| Nishiyama-2025 | Low | Low concern |
| Almalbis-2025 | High | High concern |
| Chan-2025 | Low | Low concern |


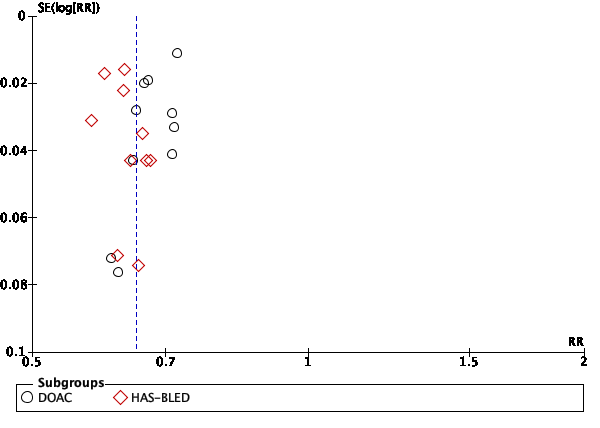


**Supplemental Figure 1.Publication bias assessed visually by funnel plots: DOAC versus HAS-BLED**


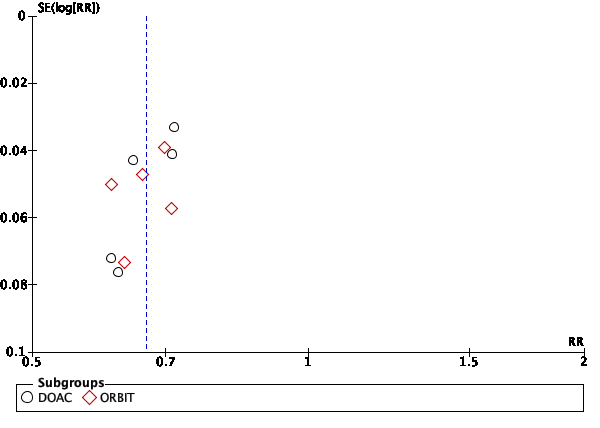


**Supplemental Figure 2.Publication bias assessed visually by funnel plots: DOAC versus ORBIT**
